# Primary* Nocardia* Infection Causing a Fluorodeoxyglucose-Avid Right Renal Mass in a Redo Lung Transplant Recipient

**DOI:** 10.1155/2018/9752860

**Published:** 2018-02-07

**Authors:** Sreeja Biswas Roy, Mitchell D. Ross, Pradnya D. Patil, Richard Trepeta, Ross M. Bremner, Tanmay S. Panchabhai

**Affiliations:** ^1^Department of Internal Medicine, St. Joseph's Hospital and Medical Center, Phoenix, AZ, USA; ^2^Department of Hematology and Oncology, Taussig Cancer Institute, Cleveland Clinic, Cleveland, OH, USA; ^3^Department of Pathology, St. Joseph's Hospital and Medical Center, Phoenix, AZ, USA; ^4^Department of Thoracic Surgery, Norton Thoracic Institute, St. Joseph's Hospital and Medical Center, Phoenix, AZ, USA; ^5^Department of Pulmonary Medicine, Norton Thoracic Institute, St. Joseph's Hospital and Medical Center, Phoenix, AZ, USA

## Abstract

Immunosuppression after lung transplantation may increase susceptibility to opportunistic infection and is associated with early and delayed deaths in lung transplant recipients. Factors that may predispose lung transplant recipients to opportunistic bacterial and fungal infections include prolonged corticosteroid use, renal impairment, treatment of acute rejection, and post-transplant diabetes mellitus. We present a unique case of a 63-year-old woman with diabetes mellitus who underwent redo lung transplantation. Three years after her right-sided single redo lung transplant, she presented with right-sided abdominal pain, nausea, and vomiting. Upon examination, computed tomography showed a 4.5 × 3.3 cm heterogeneous, enhancing right renal mass with a patent renal vein. Magnetic resonance imaging confirmed a T1/T2 hypointense, diffusion-restricting, right mid-renal mass that was fluorodeoxyglucose-avid on positron emission tomography. We initially suspected primary renal cell carcinoma. However, after a right nephrectomy, no evidence of neoplasia was observed; instead, a renal abscess containing filamentous bacteria was noted, raising suspicion for infection of the* Nocardia* species. Special stains confirmed a diagnosis of* Nocardia* renal abscess. Computed tomography of the chest and brain revealed no lesions consistent with infection. We initiated a long-term therapeutic regimen of anti-*Nocardia* therapy with imipenem and trimethoprim-sulfamethoxazole.

## 1. Introduction

In recent decades, lung transplantation has been increasingly used as a treatment for patients with end-stage lung disease [1]. Immunosuppression after lung transplantation involves triple-drug therapy: prednisone, a calcineurin inhibitor (either tacrolimus or cyclosporine), and an antimetabolite (either mycophenolate mofetil or azathioprine) [1]. These drugs increase the risk of opportunistic infections and malignancies, especially skin cancer [1]. Although lung transplant recipients have a higher incidence of solid organ tumors, they often have an atypical presentation. A high index of suspicion is required for potentially malignant lesions due to their high propensity for early metastasis.

Atypical infections such as* Nocardia* are only seen in up to 2% of lung transplant recipients [2]. Most of these patients receive trimethoprim-sulfamethoxazole (TMP-SMZ) prophylaxis for pneumocystis jiroveci pneumonia (PJP) that is also protective against* Nocardia*.* Nocardia* infection usually presents as a pulmonary nodule, a mass, or a consolidation with positive bronchoalveolar lavage (BAL) or sputum cultures [2]. Primary* Nocardia* infection of nonpulmonary sites is exceedingly rare, but dissemination to other organs can be seen, especially with augmented immunosuppression. When* Nocardia* spreads, it has a predilection for skin, soft tissue, and the brain. Such lesions often can mimic solid organ tumors or post-transplant lymphoproliferative disorder (PTLD). Although no specific guidelines exist regarding antimicrobial therapy for nocardiosis in lung transplant recipients, ongoing immunosuppression often requires prolonged multidrug therapy to reduce the chances of relapse or disease progression [2].

## 2. Case Presentation

A 63-year-old white woman diagnosed with diabetes mellitus underwent right lung transplant for bronchiolitis obliterans syndrome (BOS) three years after an original left single lung transplant for idiopathic pulmonary fibrosis. Induction immunotherapy prior to re-transplantation consisted of antithymocyte globulin (ATG) and methylprednisolone. She had also received a course of ATG for management of BOS in her initial left lung transplant. Current maintenance immunosuppression included mycophenolate mofetil, tacrolimus, and prednisone, in addition to post-transplant antimicrobial prophylaxis with TMP-SMZ, valganciclovir, and itraconazole. She had multiple episodes of Epstein-Barr viremia within the first six months after transplant. She also experienced multiple infectious complications, including pseudomonas pneumonia, enterococcal tracheobronchitis, and most recently invasive right lower lobe pulmonary aspergillosis treated with isavuconazonium sulfate two and a half years after her redo lung transplant.

She presented to the emergency department three years after her redo lung transplant experiencing fluctuating, insidious-onset right lower abdominal pain. Her symptoms had been present for ten days and were associated with nausea, vomiting, and diarrhea. Four weeks earlier, she had been prescribed oral antibiotics for a lower urinary tract infection. Upon presentation, the patient was afebrile with sinus tachycardia (heart rate: 114 beats per minute) and otherwise normal vital signs. Her chest and abdominal examinations were unremarkable. Her laboratory data were significant for mild neutrophilic leukocytosis of 14.6 × 10^6^/microL, stable microcytic hypochromic anemia of 10.4 g/dl, and stable hyponatremia of 131 mmol/L. Urinalysis showed hazy urine with few squamous cells, >50 WBC, 3–10 RBCs, 30 mg/dl protein, moderate leukocyte esterase, and 11–25 hyaline casts/LPF.

**Figure 1:**
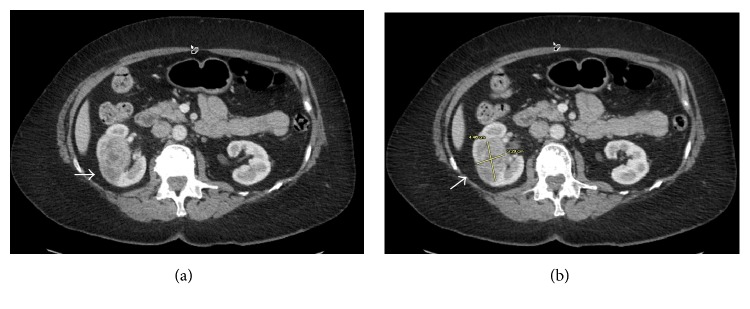
Computed tomogram of the abdomen-pelvis with contrast shows a 4.5 × 3.3 cm heterogeneous, enhancing soft-tissue density mass within the right kidney with a patent right renal vein (white arrow).

**Figure 2:**
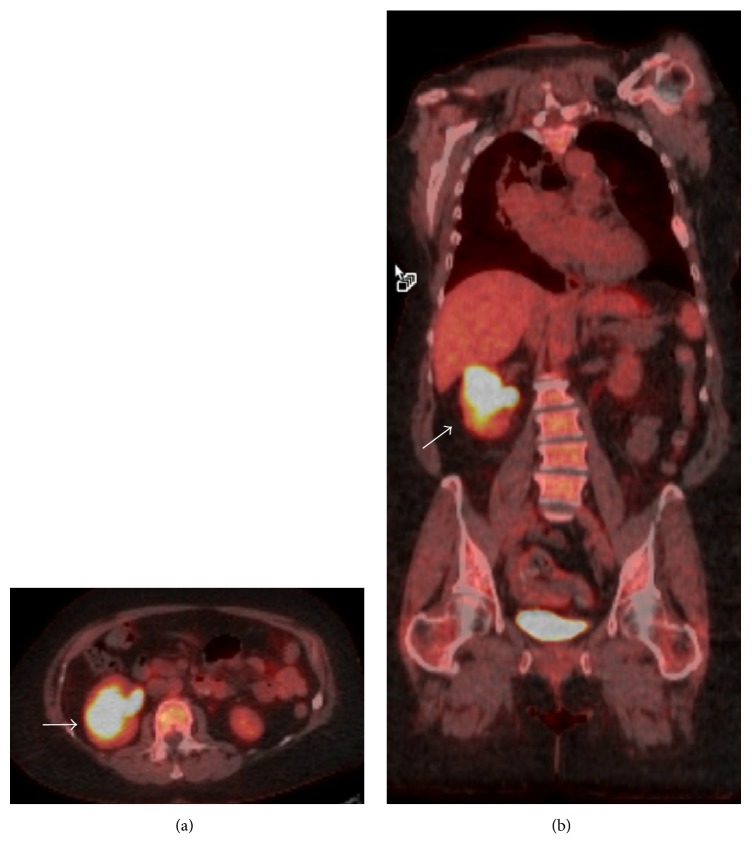
Positron emission tomography shows an intensely fluorodeoxyglucose-avid right renal mass with imaging characteristics highly suspicious for renal cell carcinoma (white arrow).

An abdominal ultrasound revealed a 3.3 × 2.8 × 3.1 cm complex, solid-appearing mass within the mid-pole of the right kidney with peripheral vascularity. This mass was not seen on an abdominal computed tomogram (CT) performed four weeks earlier. A CT with contrast confirmed the 4.5 × 3.3 cm heterogeneous, enhancing soft tissue right renal mass with patent renal vein and no retroperitoneal lymphadenopathy (Figure 1). These imaging findings raised suspicion for renal cell carcinoma (RCC) and prompted magnetic resonance imaging of the abdomen. This showed a T1/T2 hypointense, diffusion-restricting, right mid-renal mass very suspicious for RCC. The patient subsequently underwent a staging positron emission tomography-CT that showed an intensely fluorodeoxyglucose-avid right renal mass (standard uptake value = 14.3) (Figure 2).

**Figure 3:**
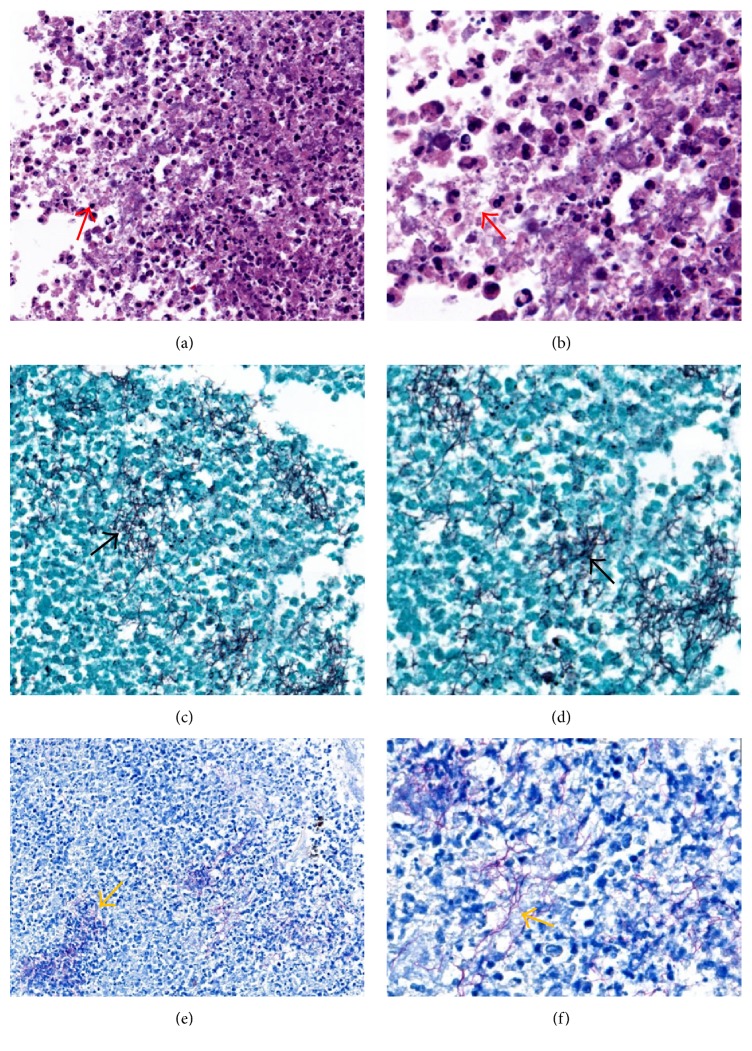
Renal mass pathology shows multiple renal abscesses with filamentous structures (red arrow) on hematoxylin and eosin stain under (a) low power (40x) and (b) high power (100x). Gomori's methenamine silver stain shows no fungal hyphae but highlights the filamentous bacterial forms (black arrow) under (c) low power (40x) and (d) high power (100x). These filamentous bacteria stain weakly positive with Fite's stain supporting the diagnosis of* Nocardia* species (yellow arrow) under low power (40x) (e) and high power (100x) (f).

The patient was then evaluated by urology and oncology and underwent a hand-assisted right nephrectomy for a presumptive pre-operative diagnosis of RCC. Histopathology revealed a blue-green necrotic renal mass measuring 5.5 × 5 × 4 cm, located in the superior lateral pole, and compressing the superior calyx without pelvicalyceal involvement. There was no evidence of malignancy, but multiple filamentous bacteria consistent with the* Nocardia* species were identified. Methenamine silver stain showed filamentous bacteria-like structures staining positive on gram stain which were weakly positive on the Fite staining method, supporting a diagnosis of* Nocardia* renal abscess (Figure 3). CTs of the head and chest revealed no lesions suspicious for nocardiosis. Because the suspicion of RCC was extremely high based on imaging, the entire renal specimen was sent for pathological analysis as a fixed specimen but not for culture. Polymerase chain reaction (PCR) for* Nocardia* species on this nephrectomy specimen revealed* N. paucivorans*. She was subsequently started on dual therapy with imipenem and double strength TMP-SMZ. Imipenem was continued for 8 weeks, and TMP-SMZ was to be continued for 9 to 12 months thereafter.

## 3. Discussion

Nocardiosis is mostly an opportunistic infection caused by Gram-positive aerobic bacteria belonging to the genus* Nocardia*. The bacteria are found ubiquitously in soil, organic matter, and water, and the lungs are the primary port of entry in the pathophysiology for* Nocardia* infection [2].* Nocardia* are notorious for causing localized or disseminated infections in both normal and immunocompromised humans. Predisposing factors for* Nocardia* infection include malignancy, organ transplant, immunosuppressed status, chronic granulomatous disease, radiation therapy, and tuberculosis [3]. These organisms can affect virtually any organ system through hematogenous spread, are a diagnostic challenge due to their varied presentation, and often result in relapse or progression of infection despite appropriate therapy. Distant metastatic involvement of the brain has been described in cases of lung infection with a* Nocardia* species [4].

Rates of* Nocardia* infection after lung transplantation range from to 1.8% to 2.1% [5, 6]. The lungs are affected in most (99%) cases reported in lung transplants, with other sites including skin, soft tissue, brain, and disseminated infection [7]. Diagnosis requires positive BAL, sputum culture, or biopsy. The lung lesions commonly appear as nodular infiltrates on radiography and CT imaging, with or without cavitation.* Nocardia* species grow slowly with cultures generally becoming positive after five days of incubation and are often not visualized on initial stains. This often delays clinical diagnosis and thwarts effective, timely antimicrobial therapy [7]. A presumptive diagnosis can be made if branching, partially acid-fast filamentous rods are identified in biopsy specimens.

Low rates of* Nocardia* infection in lung transplant recipients are in part due to the ability of TMP-SMZ used as a prophylactic against PJP [4] to also prevent* Nocardia* infection [4]. Risk factors for development of* Nocardia* infection in lung transplant recipients include inability to tolerate TMP-SMZ (which can be substituted with dapsone, atovaquone, or inhaled pentamidine) and augmented immunosuppression to treat episodes of rejection. Augmented immunosuppression can include pulse steroids and other agents, such as ATG. The clinical risk factors predisposing for primary nonpulmonary nocardiosis are not clearly understood, given the rarity of this presentation.

Differential diagnoses in cases of new lung nodules, masses, or consolidation can include other opportunistic infections such as aspergillus, mycobacteria, PTLD, and, rarely, lung cancer or metastasis from nonpulmonary solid tumors. Given the isolated fluorodeoxyglucose-avid right renal mass in our patient and the radiographic characteristics of this mass being consistent with RCC, nocardiosis was not high on the list of differential diagnoses. The lack of evidence of the renal mass on a CT scan from four weeks earlier does support a rapidly expanding infectious process, but certain aggressive tumors could present in similar fashion. Because of the high pretest probability of RCC and the small albeit real risk of seeding with biopsy [8], we elected to proceed with a right nephrectomy.

Effective antibiotics for* Nocardia* infection include TMP-SMZ, third-generation cephalosporins, extended-spectrum quinolones, carbapenems, minocycline, and linezolid. Several reports have stressed the importance of speciation due to varying antimicrobial sensitivities among* Nocardia *species. While PCR test is a useful test for the etiological diagnosis, bacteriological culture of the specimen, typing of the isolates, and antibiotic susceptibility test are also very important for the management of nocardiosis as some species of* Nocardia* can be resistant to conventional antibiotics. In our case, culture sensitivities were not sent due to the high pretest probability of RCC, so the nephrectomy specimen was sent as a fixed specimen. However, even though PCR can be used to establish species and predict antimicrobial sensitivity, culture remains the gold standard and should be sent in all patients where* Nocardia* infection is suspected. In our patient, PCR testing revealed* N. paucivorans* species.

*Nocardia* infections, although uncommon, should be considered in the differential diagnosis of nodular masses in lung transplant recipients regardless of the mass location. In extrapulmonary lesions, a rapid growth pattern can suggest (but not define) an infectious etiology. If a patient has a history of augmented immunosuppression, an atypical infection should be strongly considered in the differential diagnosis. Although recent evidence suggests that the risk of seeding with percutaneous biopsies is low, the typical clinical picture of renal abscess or lung-renal involvement with* Nocardia* may not be clear in recipients of solid organ transplants. The decision to proceed with surgical therapy is complicated, especially in immunosuppressed patients in whom solid tumor malignancies tend to spread rapidly. However, surgical specimens should always be sent for culture to determine antibiotic sensitivities accurately.
